# Comparison between absolute ethanol and bleomycin for the treatment of venous malformation in children

**DOI:** 10.3892/etm.2013.1144

**Published:** 2013-06-05

**Authors:** JING ZHANG, HAI-BO LI, SHAO-YI ZHOU, KUN-SHAN CHEN, CHUAN-QIANG NIU, XIAO-YUN TAN, YI-ZHOU JIANG, QUE-QING LIN

**Affiliations:** Department of Interventional Radiology and Vascular Anomalies, Guangzhou Women and Children’s Medical Center, Guangzhou 510120, P.R. China

**Keywords:** children, venous malformation, sclerotherapy, absolute ethanol, bleomycin

## Abstract

The aim of this study was to investigate the therapeutic efficacies and treatment effects of absolute ethanol and bleomycin for the treatment of venous malformation (VM) in children. A total of 138 children with VM were randomly divided into two groups; 75 patients were treated with absolute ethanol, while a further 63 were treated with bleomycin under general anesthesia between February 2009 and February 2012. The treatment outcome and complications were observed in the two groups and the treatment efficacy was classified as one of four categories: cured, markedly effective, effective and ineffective. The curative effect was analyzed 6–24 months after treatment, with a mean of 15 months. Absolute ethanol was effective (cured, markedly effective or effective) in 71 cases and bleomycin was effective in 41 cases, and the difference between the effective rates was considered to be statistically significant (χ^2^=19.6, P<0.05). In the absolute ethanol group there were 14 cases with skin necrosis, 17 patients had serious localized swelling which required additional treatment, three patients developed muscle fibrosis and one patient suffered a brain embolism. In the bleomycin group there were five cases with skin necrosis and the difference in the incidence of adverse reactions was considered to be statistically significant (χ^2^=18.8, P<0.05). The curative effect of sclerotherapy for VM is clear, and absolute ethanol is the most effective sclerosing agent, but has a greater incidence of adverse side-effects than bleomycin. The major side-effect is skin necrosis. The choice of sclerotherapy depends on the classification of VM in children.

## Introduction

Venous malformation (VM) is a common vascular anomaly in children (1,2), and is mainly composed of abnormally dilated venous components without cell proliferation characteristics. Compared with other vascular malformations, the type, location, scope and tissue invasion of VM are significantly diverse. There are various treatments for VM, including surgical excision, laser therapy and interventional sclerotherapy. Previously, surgical resection had been considered to be the preferential treatment (3). However, the drawbacks of surgical resection are clear: it is not possible to remove the tumors completely, recurrence after surgery is common, surgical trauma is serious and the peripheral nerve may be injured. The effects of laser therapy are limited and this technique is only effective in patients with small superficial tumors. Recently, interventional sclerotherapy has become the main method of treatment (4,5). Common sclerosing agents include foam sclerosing agents, polidocanol, sodium tetradecyl sulfate, anhydrous ethanol and bleomycin. Anhydrous ethanol is the most widely used sclerosing agent. There are numerous reports regarding treating VM with a single dose of anhydrous ethanol or bleomycin (6–8). However, few reports provide a comprehensive comparison of treatment effects and adverse reactions between the two methods. Between February 2009 and February 2012, 138 children with VM were treated with either anhydrous ethanol or bleomycin. This study was designed in order to compare the treatment effects and adverse reactions of anhydrous ethanol and bleomycin and to provide evidence for the selection of sclerosing agents.

## Patients and methods

### Patients

All 138 patients with VM in this study were diagnosed in accordance with the diagnostic criteria of VM in Clinical Practice Guidelines - Plastic Surgery Volume (9): the lesions existed at birth; the lesions grew in proportion with the body; when the focus location was superficial, the lesion area was blue; the texture was soft and compressible; the skin temperature in the lesion area was not high (a key point for hemangioma identification); and it tested positive in postural experiments. Also, characteristic manifestations were observed by MRI. This study involved 138 patients, 83 males and 55 females, aged between 3 months and 14 years. Signed informed consent was obtained from guardians of all patients. The study was approved by the ethics committee of Guangzhou Women and Children’s Medical Center.

### Medical history

The foci of 112 patients existed when they were born and grew as the patients did; the foci of 26 patients occurred 3 months after birth. In 83 cases (60%), the tumors were located in the maxillofacial region, in 28 cases (20%) the tumors were located in the limbs, in 17 cases (12%) the tumors were located in the trunk and in 10 cases (7%) the tumors were located in the hip. There were 87 cases of superficial VM; in 55 cases the foci were located in the subcutaneous tissues and of those, 22 were located in the mucous membrane. There were 51 cases of deep VM with the foci located in deep muscle tissue. Foci involving skin or mucosal surfaces were bluish-violet in color and were raised from the skin surface or mucous membranes; the foci located in deep muscle tissue were expressed as masses. The texture of the masses was soft and tested positive in a postural experiment. The tumor size ranged from 2.5×2.0×1.0 cm to 15.0×10.0×6.0 cm. There were 66 patients whose chief complaint of irregular pain at the site of the lesion could be relieved without further treatment. All patients were examined using MRI (Philips Achieva 1.5T dual-gradient magnetic resonance imager; Royal Philips Electronics, Amsterdam, The Netherlands). The lesion demonstrated a low or iso-signal on the T1-weighted image (T1WI) and a high signal on the T2WI. The enhanced scan revealed 39 cases of marked enhancement, 62 cases of mild to moderate enhancement and 37 cases of no enhancement. Using ultrasound, the lesion was expressed as an uneven internal echo with clear boundary and irregular shape and a pipe-like echo was visible. Blood flow was not probed by a Doppler test. All surgeries were carried out under the guidance of the large C-arm angiography machine (GE, Waukesha, WI, USA). Drugs used during surgery included bleomycin injection (8 mg/tube; Harbin Lebo Pharmaceutical Co., Ltd., Harbin, China), lipiodol injection (10 ml/tube; Guerbet, Aulnay-sous-Bois, France) and iohexol injection (20 ml, 6 mg: GE Pharmaceutical Co., Ltd., Shanghai, China).

### Treatment method

The 138 patients with VM were randomly divided into group A and group B according to hospitalization number and were selected on the basis of admission time. Patients whose hospitalization number ended in an odd number were assigned to group A with anhydrous ethanol and lipiodol as the sclerosing agent; patients whose hospitalization number ended in an even number were assigned to group B with bleomycin and lipiodol as the sclerosing agent. The ratio of anhydrous ethanol to lipiodol in the sclerosing agent of group A was 5:1 (v/v). The maximum dosage of anhydrous ethanol was 1 mg/kg, with a single dosage ≤50 ml. When the dosage of anhydrous ethanol was assumed to exceed 0.5 ml/kg, pulmonary artery pressure was monitored. To prepare the sclerosing agent of group B, 8 mg bleomycin was dissolved in 4 ml contrast agent; the dosage of bleomycin was calculated at 10 mg/m^2^ body surface. The solution was mixed with an equal amount of ultra-liquefied lipiodol in a sterile container and the mixture was aspirated repeatedly with a syringe to prepare a bleomycin-lipiodol emulsion. The concentration of bleomycin was 1 mg/ml.

Due to the poor cooperation of children and the irritating nature of the sclerosing agents, all patients were treated under general anesthesia. After general anesthesia, the malformed vascular mass was directly punctured for radiography. The most protuberant part of the VM was punctured directly using 7th scalp acupuncture (Venofix (r) A). When the malformed vascular mass was successfully punctured, venous blood was pumped back. Then, 30% (iodine content) contrast agent (iohexol injection) was injected under fluoroscopy and the filling situation of the VM was continuously observed. Prior to treatment with a sclerosing agent, the patients were injected intramuscularly with dexamethasone at a dose of 0.3 mg/kg. In group A, anhydrous ethanol-lipiodol emulsion was injected into abnormal vessels; in group B, patients were treated with a bleomycin-lipiodol emulsion. Prior to treatment, the proximal-end draining veins of the VM patients in the two groups were pressed directly by assistants to expand the malformed vascular mass. The sclerosing agent was injected slowly into the malformed vascular mass under fluoroscopy via scalp acupuncture which was used for injection of the contrast agent. During the injection process, embolization agents were carefully observed to monitor whether they entered the draining veins and assess the filling situation of the vessel mass. When the vessel mass or the draining vein was shown to be filled completely, the injection of sclerosing agent was stopped. If the malformed vascular mass was unable to be filled completely via one injection site, another puncture site was used to inject the embolization agent. Patients who were cured were followed up. If tumors were reduced by <80%, the treatment was continued. The time interval between treatments was one month.

### Efficacy criteria and follow-up

All patients were reviewed one month after treatment and the efficacy was evaluated. If patients with superficial VM, whose efficacy could be judged by the naked eye, were cured, they were followed up. If the tumors were reduced by <80%, treatment was continued. For deep VM, efficacy was evaluated by MRI examination. The final efficacy was the result of an MRI follow-up visit carried out 6 months after the final treatment. The treatment efficacy was classified into four levels (7): i) cured, the tumors completely disappeared after injection, the surface color was normal, no dysfunction and no recurrence during the follow-up visit. ii) Markedly effective, the majority of the tumors disappeared after injection (tumors were reduced by >80%), skin color was close to normal or there was slight pigmentation, no dysfunction, the appearance had not yet completely recovered and the treatment should be continued. iii) Effective (improved), tumors were reduced by <80% and the treatment should be continued. iv) Ineffective, tumors were not reduced; they remained unchanged or continued to increase. The effective rate = (the number of cured cases + the number of markedly effective cases + the number of effective cases) / the number of total cases × 100. Systemic and local adverse reactions of the patients were recorded. Since all patients demonstrated swelling and pain, which was relived without treatment 3–7 days after surgery, and swelling was a part of the sclerotherapy mechanism, swelling and pain were not classified as adverse reactions.

### Statistical analysis

SPSS 13.0 (SPSS, Inc., Chicago, IL, USA) was used for statistical analysis. The comparison of effective rates and incidence of adverse reactions between the two groups were tested using the χ^2^ test. P<0.05 was considered to indicate a statistically significant difference.

## Results

### Efficacy

**Figure 1.**
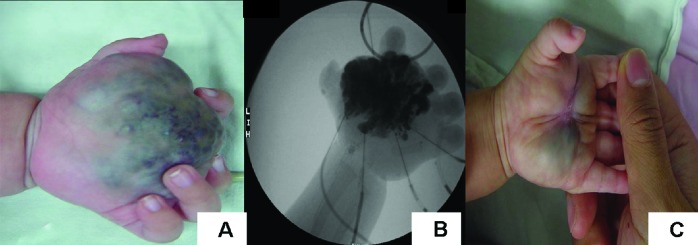
The patient in these images was 10 months old when treated. When the patient was born, a tumor was discovered on the left hand, which gradually increased in size. (A) The patient was diagnosed with venous malformation. (B) Percutaneous interventional sclerosing therapy was guided by MRI. During surgery 6 mg bleomycin, 3 ml iohexol and 3 ml ultra-liquefied lipiodol were used. The patient was treated with interventional sclerosing therapy a total of three times. (C) The patient was reviewed 6 months after surgery and the size of the tumor had reduced.

**Figure 2.**
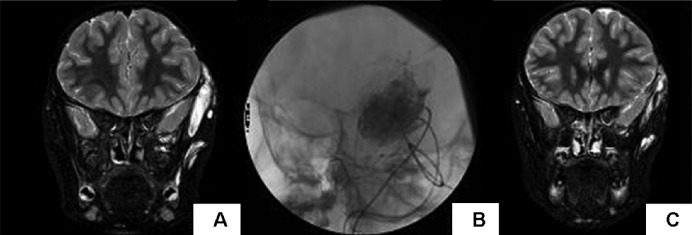
The patient in these images was 1 year old. (A) Venous malformation was located in the left maxillofacial region. (B) Percutaneous interventional sclerosing therapy was guided by MRI. During surgery, 10 ml anhydrous ethanol and 2 ml ultra-liquefied lipiodol suspension were used. (C) The patient was treated once and reviewed after 5 months and the tumors were found to be reduced in size.

**Figure 3.**
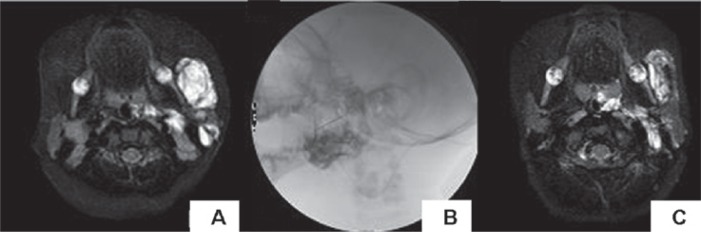
The patient in these images was 8 years old. (A) Venous malformation was located in the left tempus; (B) Percutaneous interventional sclerosing therapy was guided by MRI. During surgery 5 mg bleomycin, 2.5 ml iohexol and 2.5 ml ultra-liquefied lipiodol suspension were used; the patient was treated twice. (C) The patient was reviewed two months after surgery and the tumors were reduced in size.

The patients in the two groups were followed up for 6–24 months. The observed efficacy is demonstrated in Table I. In group A, the effective rate of superficial VM was 95% (38/40) and that of deep VM was 94% (33/35). In group B, the effective rate of superficial VM was 68% (32/47) and that of deep VM was 56% (9/16). The difference in the effective rate between the two groups was considered to be statistically significant. The efficacy rate in group A was greater than that in group B. In group A, 30 cases were treated once with anhydrous ethanol embolotherapy, 30 cases were treated twice and 15 cases were treated three times. In group B, 6 cases were treated once with bleomycin embolotherapy, 23 cases were treated twice, 31 cases were treated three times and three cases were treated four times (Table II; Figs. 1–3).

### Adverse reactions

The incidence adverse reactions was relatively high in the patients in group A (45%). In this study, six patients (two cases of superficial VM and four cases of deep VM) contracted a fever with nausea and vomiting after surgery, and the symptoms were relieved with treatment. Skin necrosis occurred in 14 superficial VM patients, and a scar remained after scabbing. There were 17 patients with serious localized swelling which required additional treatment, three patients developed muscle fibrosis and one patient suffered a brain embolism. The symptoms were relieved once the micro-circulation was improved by the intravenous infusion of *Salvia miltiorrhiza* and low molecular weight dextran. Muscle injury and fibrosis occurred in three cases of deep VM, leading to clubfoot. Cerebral infarction occurred in one case of deep VM located in the neck. The manifestations were hyperspasmia and vomiting. No sequelae remained after symptomatic treatment. The incidence of patients with an adverse reaction in group B was relatively low (10%). Fever accompanied by vomiting occurred in six patients. There were two patients (one case of deep VM and one case of superficial VM) who demonstrated swelling and tumor pain. The symptoms were relieved after treatment. A further five patients suffered from skin ulceration. The difference in the incidence of adverse reactions between the two groups was considered to be statistically significant (χ^2^=18.8, P=0.0001).

## Discussion

Sclerotherapy is the first-line therapy for VM. However, the guidelines for the selection of sclerosing agents were considered to be insufficient. Anhydrous ethanol and bleomycin are liquid sclerosing agents, but it is unclear which one should be used preferentially, as reports offer differing conclusions. It has been reported that the effective rate of treatment for VM with anhydrous ethanol was 75–95% (10–13). In this study, the effective rate of embolotherapy with anhydrous ethanol for superficial VM was 95% and for deep VM was 94%; the total effective rate was 95%. It has been reported that the effective rate of treatment for VM with bleomycin was 82.7% (14). In the current study the effective rate of treatment with bleomycin for superficial VM was 68% and for deep VM was 56%, with a total effective rate of 65%. The efficacy of embolotherapy with anhydrous ethanol was greater than that with bleomycin. However, the dehydration and denudation effects of anhydrous ethanol were more serious and the adverse reaction rate of anhydrous ethanol was high, particularly in the treatment of superficial VM. It was reported by Berenguer *et al* (15) that the rate of adverse reaction to embolotherapy with anhydrous ethanol was 50% for VM. The majority of adverse reactions involved vesicle tension and ulcer and nerve injury. Of the serious adverse reactions, skin necrosis occurred in 14 patients and cerebral infarction occurred in one patient. The dosage of anhydrous ethanol in patients who developed serious adverse reactions exceeded 0.5 ml/kg. Generally, anhydrous ethanol was injected into the draining vein. If there was no further lesion increase, the treatment was ended. If the dosage was excessive, the anhydrous ethanol would flow back to the feeding artery, leading to skin and tissue necrosis (5,11,16). Therefore, it is essential that the dosage of anhydrous ethanol is selected on the basis of tumor size.

Compared with anhydrous ethanol, bleomycin has a longer shelf life and a lower price. However, the effect of bleomycin is mild. Severe pain does not occur following the injection of bleomycin and there are fewer adverse reactions. It has been reported that the main adverse reaction of bleomycin is pulmonary fibrosis. However, in previous studies (17), pulmonary fibrosis did not occur when bleomycin was the sclerosing agent, and severe adverse reactions were rare. The majority of adverse reactions involved an increase in body temperature and localized swelling. In this study, fever and vomiting occurred in six patients in group B, two of whom demonstrated swelling and tumor pain. A further five patients developed skin ulceration. The incidence of adverse reaction was 10%.

At present, there is no consensus on the selection of sclerosing agents for clinical applications. Large scale randomized clinical trials are insufficient. Most clinicians select sclerosing agents according to their own familiarity with sclerosing agents and the focus size. Although there have been many reports (18–22) regarding treatment of VM with various sclerosing agents, the efficacy of anhydrous ethanol has rarely been compared with that of bleomycin. In the current study, it was demonstrated that the efficacy of anhydrous ethanol was greater than that of bleomycin. Although treatment with anhydrous ethanol was effective and fast, the incidence of adverse reactions was higher. For superficial VM, the efficacy of bleomycin emulsion is greater, with fewer adverse reactions. For deep VM, anhydrous ethanol is more effective.

## Figures and Tables

**Table I.** Effect of interventional therapy on the two groups.

Group	Cases	Evaluation of therapeutic effect			

		Cured	Markedly effective	Effective	Ineffective
A	75	15	33	13	4
B	63	6	19	16	22
χ^2^ value	19.6				
P-value	0.0001				

Group A, anhydrous ethanol-lipiodol emulsion embolotherapy group; group B, bleomycin-lipiodol emulsion embolotherapy group.

**Table II.** Therapeutic effect of local injection treatment for venous malformation.

Number of treatments	Group A					Group B					χ^2^ value	P value
	
	Cases	Cured	Markedly effective	Effective	Ineffective	Cases	Cured	Markedly effective	Effective	Ineffective		
1	75	9	30	28	8	63	3	12	21	27	18.7	0.0001
2	45	4	20	15	6	57	2	15	8	32	19.7	0.0001
3	15	2	9	0	4	34	1	5	6	22	6.04	0.01
4	0	0	0	0	0	3	0	3	0	0		`

Group A, anhydrous ethanol lipiodol emulsion embolotherapy group; group B, bleomycin lipiodol emulsion embolotherapy group.
